# Alcohol consumption has a J-shaped association with bacterial infection and death due to infection, a population-based cohort study

**DOI:** 10.1038/s41598-025-90197-8

**Published:** 2025-03-01

**Authors:** Karl Stattin, Mikael Eriksson, Robert Frithiof, Rafael Kawati, Douglas Crockett, Michael Hultström, Miklos Lipcsey

**Affiliations:** 1 Department of Surgical Sciences, Anesthesiology and Intensive Care, Uppsala University, Akademiska sjukhuset, ingång 70, 751 85 Uppsala, Sweden; 2 Nuffield Department of Clinical Neurosciences, University of Oxford, Oxford, UK; 3 Department of Medical Cell Biology, Integrative Physiology, Uppsala University, Uppsala, Sweden; 4 Hedenstierna Laboratory, Department of Surgical Sciences, Uppsala University, Uppsala, Sweden

**Keywords:** Alcohol, Sepsis, Bacterial infection, Mortality, Intensive care, Diseases, Risk factors, Public health

## Abstract

The aim of this study is to investigate the association between alcohol consumption and the risk of bacterial infection and its dose–response association. Participants in the Swedish Mammography Cohort and Cohort of Swedish Men answered lifestyle questionnaires in 1997 and have since been followed in national registers. The risks of acquiring infection, intensive care unit (ICU) admission and dying due to infection were assessed with Cox regression. Among 58,078 cohort participants followed for 23 years, 23,035 participants were diagnosed with an infection and 4,030 died from infection. Alcohol consumption exhibited a J-shaped association with the risk of acquiring infection and dying due to infection: compared to consuming 5–10 g of alcohol per day, consuming < 0.5 g/day and consuming > 30 g/day were both associated with higher risk of acquiring infection, ICU admission and dying due to infection, whereas alcohol consumption between 5 and 30 g/day was not associated with acquiring infection, ICU admission or death due to infection. In conclusion, moderate alcohol consumption was not associated with infection, but both very low and high levels of consumption were associated with acquiring infection, ICU admission and death. If replicated, this suggests that reduction of alcohol consumption might reduce mortality from bacterial infections.

## Introduction

Infectious disease remains one of the most important causes of disability and death worldwide^1^. Severe infection can lead to sepsis, a dysregulated immune response that can cause life-threatening organ dysfunction and potentially need for intensive care^2^. With widely adopted vaccination programs, readily available clean water and safe food sources, life-style risk factors such as alcohol consumption are quickly emerging as the most important modifiable risk factors in high-income countries. However, the association between alcohol consumption and the risk of bacterial infection is incompletely understood.

Alcohol consumption negatively affects the immune system,^3,4^ and previous studies indicate that alcohol abuse may be a risk factor for acquiring^5–11^ and dying^12–14^ due to bacterial infection. Further, alcohol abuse has been implicated as an important disease trajectory towards death due to sepsis^15^. However, most studies only investigate pneumonia, and do not examine other bacterial infections,^5–9,12,13,16^, and a number of studies have failed to replicate these findings^16–18^. Further, interpretation of the data is hampered as most previous studies have dichotomized individuals into either high or low consumption,^6–8,10–12,14,17^ meaning moderate consumption is not studied and possible nonlinear dose–response relations are not explored. Moreover, the amount classified as heavy drinking has often been very high, e.g. > 200 g per week or diagnosed alcohol abuse^6,8,14,18^. The few studies investigating the full range of alcohol consumption and developing bacterial infection have either found no association^16^ or higher risk only in individuals with very high consumption^5,6^.

Hence, the association between the full range of alcohol consumption and the risk of developing and dying due to all types of bacterial infection is not known. The aim of this study is thus to investigate the association between alcohol consumption and developing bacterial infection or pneumonia, intensive care unit (ICU) admission due to bacterial infection or pneumonia and dying due to bacterial infection or pneumonia. The full spectrum of alcohol consumption will be investigated, searching for nonlinear dose–response relations and possible threshold values.

## Methods

The National Ethical Review Agency, Stockholm, Sweden, has approved this study (DNR 2022-05751-01). The Helsinki declaration and the STROBE statement were followed^19^.

Participants were drawn from the population-based cohorts Swedish Mammography Cohort (SMC) and the Cohort of Swedish Men (COSM), which invited residents in Sweden by mail. SMC invited all women living in Uppsala county born 1914–1948 and all women living in Västmanland county born 1917–1948 to answer a lifestyle questionnaire in 1987, which was repeated in 1997. COSM invited all men living in Västmanland and Örebro counties born 1918–1952 to answer a similar questionnaire in 1997. Response rates for the 1997 questionnaires was 70% for SMC and 49% for COSM, yielding 38,984 women and 45,906 men after exclusion of individuals having entered an incorrect Personal Identification Number or having been diagnosed with cancer (except non-melanoma skin cancer) or died before baseline on January 1st 1998. See cohort flowchart in Harris et al.^20^. SMC and COSM are representative of the underlying Swedish population^20,21^.

Lifestyle questionnaires answered in 1997 provided exposure and covariate information. Questions pertained to demographic information, such as marital status and education; physical activity, such as walking and exercise; smoking status; and alcohol consumption. Alcohol consumption was ascertained in a series of questions inquiring about frequency of consumption of beer, wine and spirits and amount consumed per occasion of consumption, which was summarised into grams per day. The full questionnaire is available at www.simpler4health.se and has been validated^20^.

Using the Swedish Personal Identification Number, participants were followed after cohort inception in national Swedish registers, including the National Patient Register, the Swedish Intensive Care Registry and Cause of Death Register. The National Patient Register registers all inpatient care from 1987 and all specialised outpatient care from 2001 in Sweden with complete capture and high validity^22^. The Swedish Intensive Care Registry started in 2001 and having gradually increased coverage, since a decade it includes nearly all Swedish intensive care units and records intensive care admission, physiological derangement at admission (assessed using Simplified Acute Physiology Score 3, SAPS3^23^), and treatment during ICU stay. The Cause of Death Register records underlying and contributing causes of death, as well as time of death.

A list of bacterial infections relevant to Swedish ICUs was compiled, as previously,^24,25^ and diagnoses were collected from the National Patient Register and death due to bacterial infection, both as the underlying and contributing cause of death, was garnered from the Cause of Death Register, using International Statistical Classification of Diseases and Related Health Problems (ICD-10) codes for: abdominal infections (K35, K570, K572, K574, K578, K630, K631, K65, K800, K801, K803, K804, K81, K830, K85); central nervous system infections (A39, G00, G01, G02, G039, G050, G06, G079); endocarditis (I33, I39); pneumonia(J13, J14, J15, J16, J17, J18, J85, J86); soft tissue infections (A46, M00, M01, M726); tuberculosis (A15, A16, A17, A18, A19); urogenital infections (N10, N12, N136, N70, N390); and other infections (A32, A40, A41, A42, A48, A49, B95, B96, D65, T802, R651, R572).

### Statistical analysis

Descriptive data are presented as numbers (percentages) and median (interquartile range, IQR), as appropriate. Using Cox proportional hazards regression, hazard ratio (HR) and 95% confidence interval (CI) was calculated for the risk of developing bacterial infection, ICU admission and dying due to bacterial infection; first for any bacterial infection and second for pneumonia. Individuals were followed from January 1st 1998 until first bacterial infection, death or December 31st 2021, whichever occurred first. Alcohol consumption was divided into strata (< 0.5 (including abstainers), 0.5–5, 5–10, 10–15, 15–20, 20–25, 25–30, > 30 g per day) to allow any shape of association, with the median as the reference. Confounders of interest were selected using a Directed Acyclic Graph-based approach (Supplemental Fig. 1)^26^. First, a minimally adjusted model was used, adjusting for attained age (as time scale) and sex (man, woman). Second, a fully adjusted model was applied, adjusting for attained age (as time scale), sex (man, woman), exercise (< 1 h/week, 1 h/week, 2–3 h/week, 4–5 h/week, > 5 h/week), walking or bicycling (hardly ever, < 20 min/day, 20–40 min/day, 40–60 min/day, > 60 min/day), education (< 9 years, 9–12 years, > 12 years, other), marital status (cohabiting, not cohabiting), smoking status (current, former, never) and Charlson’s weighted comorbidity index (continuous)^27^. Departure from linearity was assessed using a test of trend. Log–log-plots were used to visually assess the assumption of proportionality.

In the baseline cohort, 16.3% had missing information on alcohol consumption, 11.0% on exercise, 9.1% on walking or bicycling, 6.6% on marital status and 1.8% on smoking status. Data on emigration was not available, but emigration from Sweden was low (0.3–0.6% per year) during this period of time^28^. Missing information for all other variables was < 1%, leaving 58,078 individuals with all prerequisite information to be included in the main complete-case analysis. A number of sensitivity analyses were performed: (1) including only the underlying cause of death; (2) adjusting the analyses of any bacterial infection and pneumonia for self-rated health; (3) repeating the analysis of any bacterial infection separately by grams of alcohol from beer, wine and spirits per day; (4) repeating the analysis stratified on sex.

All analyses were performed on Stata 15.1 (Stata Corp., College station, Texas, US).

## Results

Cohort participants had a median age of 59 years (IQR 53–68) at baseline and 39.6% were women. The median alcohol consumption was 7.5 g/day (IQR 3.0–15.2), and 23.2% exhibited harmful consumption, i.e. 14 UK units (112 g) or more per week^29^. Individuals with higher alcohol consumption were younger, healthier, more often male, had higher education, were more often smokers and were less likely to live alone. Participant characteristics are shown in Table 1. During the 23 years of follow-up, comprising 1,140,880 person-years at risk, 58,078 individuals were followed, of which 23,035 suffered at least one episode of bacterial infection, 9,575 suffered at least one episode of pneumonia, and 4,030 died from bacterial infection and 2,553 died from pneumonia. The median time to event from study start was 12.9 years for acquiring infection, 13.9 years for acquiring pneumonia, 14.8 years for dying in infection and 14.4 years for dying in pneumonia.

**Table 1:** Participant characteristics by alcohol consumption.

	Alcohol consumption, grams per day							
	< 0.5	< 0.5–5	5–10	10–15	15–20	20–25	25–30	> 30
No, (%)	2,904 (5.0)	19,041 (32.8)	13,185 (22.7)	8,245 (14.2)	5,256 (9.0)	3,203 (5.5)	1,980 (3.4)	4,264 (7.3)
Age in 1997, years median (IQR)	65.0 (56.0–72.0)	62.0 (54.0–70.0)	59.0 (53.0–67.0)	58.0 (52.0–66.0)	57.0 (52.0–65.0)	56.0 (51.0–64.0)	56.0 (51.0–63.0)	56.0 (51.0–64.0)
Alcohol, grams/day median (IQR)	0.3 (0.2–0.4)	2.4 (1.4–3.6)	7.3 (6.1–8.6)	12.2 (11.0–13.6)	17.2 (16.1–18.5)	22.1 (21.0–23.4)	27.1 (26.0–28.5)	41.0 (34.1–54.5)
Women, n (%)	2,174 (74.9)	10,404 (54.6)	55,24 (41.9)	2,515 (30.5)	1,200 (22.8)	534 (16.7)	254 (12.8)	374 (8.8)
Education, n (%)								
< 9 years	1,365 (47.0)	7,647 (40.2)	4,370 (33.1)	2,409 (29.2)	1,540 (29.3)	863 (26.9)	530 (26.8)	1,228 (28.8)
9–12 years	99 (3.4)	947 (5.0)	879 (6.7)	707 (8.6)	482 (9.2)	306 (9.6)	191 (9.6)	420 (9.8)
> 12 years	350 (12.1)	2,852 (15.0)	2,587 (19.6)	1,817 (22.0)	1,173 (22.3)	726 (22.7)	463 (23.4)	908 (21.3)
Other	1,090 (37.5)	7,595 (39.9)	5,349 (40.6)	3,312 (40.2)	2,061 (39.2)	1,308 (40.8)	796 (40.2)	1,708 (40.1)
Smoking, n (%)								
Never	1,750 (60.3)	9,513 (50.0)	5,389 (40.9)	2,839 (34.4)	1,593 (30.3)	919 (28.7)	504 (25.5)	860 (20.2)
Former	609 (21.0)	5,461 (28.7)	4,689 (35.6)	3,295 (40.0)	2,288 (43.5)	1,377 (43.0)	919 (46.4)	1,847 (43.3)
Current	545 (18.8)	4,067 (21.4)	3,107 (23.6)	2,111 (25.6)	1,375 (26.2)	907 (28.3)	557 (28.1)	1,557 (36.5)
Living alone, n (%)	912 (31.4)	4,123 (21.7)	2,151 (16.3)	1,214 (14.7)	762 (14.5)	466 (14.5)	288 (14.5)	876 (20.5)
Charlson’s weighted comorbidity index								
0	2,237 (77.0)	15,494 (81.4)	11,168 (84.7)	7,146 (86.7)	4,518 (86.0)	2,793 (87.2)	1,732 (87.5)	3,646 (85.5)
1	369 (12.7)	2,055 (10.8)	1,274 (9.7)	715 (8.7)	506 (9.6)	284 (8.9)	169 (8.5)	436 (10.2)
2	225 (7.7)	1,108 (5.8)	556 (4.2)	284 (3.4)	167 (3.2)	91 (2.8)	64 (3.2)	126 (3.0)
≥ 3	73 (2.5)	384 (2.0)	187 (1.4)	100 (1.2)	65 (1.2)	35 (1.1)	15 (0.8)	56 (1.3)
Exercise, n (%)								
< 1 h/week	650 (22.4)	3,754 (19.7)	2,518 (19.1)	1,615 (19.6)	1,039 (19.8)	694 (21.7)	435 (22.0)	1,179 (27.7)
1 h/week	630 (21.7)	4,060 (21.3)	2,819 (21.4)	1,803 (21.9)	1,090 (20.7)	635 (19.8)	460 (23.2)	766 (18.0)
2–3 h/week	914 (31.5)	6,250 (32.8)	4,518 (34.3)	2,743 (33.3)	1,725 (32.8)	1,045 (32.6)	590 (29.8)	1,223 (28.7)
4–5 h/week	352 (12.1)	2,356 (12.4)	1,669 (12.7)	1,021 (12.4)	673 (12.8)	387 (12.1)	240 (12.1)	503 (11.8)
> 5 h/week	358 (12.3)	2,621 (13.8)	1,661 (12.6)	1,063 (12.9)	729 (13.9)	442 (13.8)	255 (12.9)	593 (13.9)
Walking/bicycling, n (%)								
Hardly ever	356 (12.3)	2,162 (11.4)	1,410 (10.7)	899 (10.9)	576 (11.0)	400 (12.5)	289 (14.6)	738 (17.3)
< 20 min/day	572 (19.7)	3,944 (20.7)	2,963 (22.5)	1,954 (23.7)	1,335 (25.4)	793 (24.8)	506 (25.6)	1,082 (25.4)
20–40 min/day	912 (31.4)	6,150 (32.3)	4,339 (32.9)	2,709 (32.9)	1,687 (32.1)	1,021 (31.9)	550 (27.8)	1,217 (28.5)
40–60 min/day	531 (18.3)	3,340 (17.5)	2,258 (17.1)	1,354 (16.4)	814 (15.5)	513 (16.0)	304 (15.4)	590 (13.8)
> 60 min/day	533 (18.4)	3,445 (18.1)	2,215 (16.8)	1,329 (16.1)	844 (16.1)	476 (14.9)	331 (16.7)	637 (14.9)

**Fig. 1:**
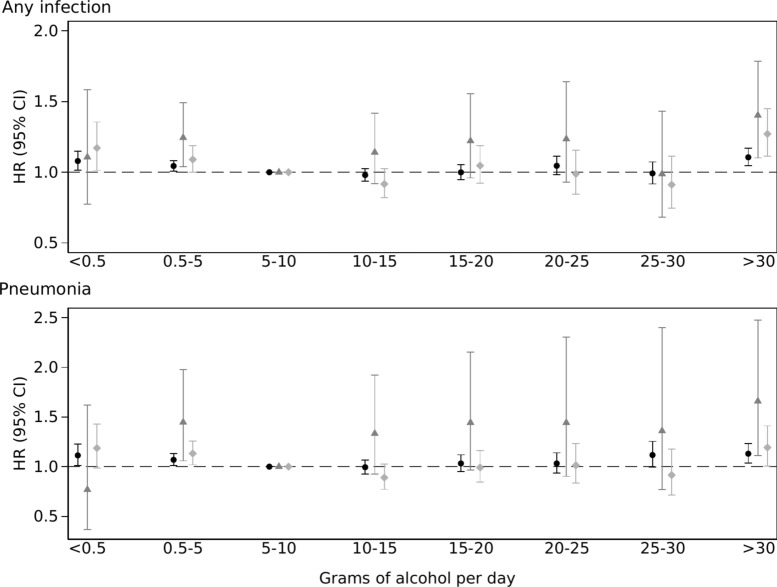
Hazard ratio (HR) and 95% confidence intervals (CI) of developing bacterial infection, ICU admission and dying due to bacterial infection by alcohol consumption. Hazard ratio (HR) and 95% confidence interval (CI) of developing bacterial infection (black circle), being admitted to an intensive care unit (grey triangle) and dying due to bacterial infection (light grey diamond) for any bacterial infection (upper panel) and pneumonia (lower panel) by alcohol consumption in grams per day, adjusted for age, sex, exercise, walking or bicycling, education, marital status, smoking status and Charlson’s weighted comorbidity index.

Compared to consuming 5–10 g/day, participants consuming < 0.5 g/day had HR 1.08 (95% CI 1.01–1.15) of acquiring bacterial infection; participants in strata between 5 and 30 g/day demonstrated no difference compared to the reference; whereas participants consuming > 30 g/day had HR 1.11 (95% CI 1.05–1.17). Compared to consuming 5–10 g/day, the risk of ICU admission in participants consuming < 0.5 g/day was HR 1.11 (95% CI 0.77 -1.58), whereas the risk in participants consuming > 30 g/day was HR 1.40 (95% CI 1.10–1.79). The risk of dying among participants consuming < 0.5 g/day was HR 1.17 (95% CI 1.01–1.36) and the risk among participants consuming > 30 g/day was HR 1.27 (95% CI 1.11–1.45) (Fig. 1, Supplemental Table 1). Tests of trend indicated departure from linearity in all outcomes.

Compared to consuming 5–10 g/day, the risk of acquiring pneumonia was higher in participants consuming < 0.5g/day, HR 1.11 (95% CI 1.01–1.23), and in participants consuming > 30 g/day, HR 1.13 (95% CI 1.04–1.23). The corresponding risks of ICU admission among participants consuming < 0.5 g/day was HR 0.77 (95% CI 0.37–1.62) and the risk among participants consuming > 30 g/day was HR 1.66 (95% CI 1.11–2.47). The risk of death among participants consuming < 0.5 g/day was HR 1.19 (95% CI 0.99–1.43), and the risk among participants consuming > 30 g/day HR 1.19 (95% CI 1.01–1.41) compared to participants consuming 5–10 g/day (Fig. 1, Supplemental Table 1). Tests of trend indicated departure from linearity in all outcomes.

Sensitivity analyses including only the underlying cause of death had similar patterns as the main analysis but wider confidence intervals and only demonstrated statistically significant higher risk of dying due to any bacterial infection in individuals consuming > 30 g/day (Supplemental Fig. 2). Analyses adjusted for self-rated health showed similar trends as the main analysis, albeit with attenuated estimates (Supplemental Fig. 3). Analyses separated by type of alcohol show no clear differences between alcohol types (Supplemental Fig. 4). Analyses stratified on sex demonstrate similar results as the main analysis, albeit more pronounced in women and with wider confidence intervals (Supplemental Fig. 5).

## Discussion

In this population-based cohort study, alcohol consumption both below 5 and above 30 g per day was associated with a higher risk of acquiring any bacterial infection or pneumonia and dying due to infection or pneumonia compared to moderate alcohol consumption. The associations were nonlinear with J-shaped dose–response curve. The hazard ratios for acquiring and dying due to bacterial infection was comparable to previous studies comparing the risk between individuals with and without diabetes^30^.

Previous studies of alcohol and bacterial infection have mainly investigated pneumonia^5–9,12,13,16^ and the majority find a higher risk of acquiring^5–11^ and dying^12,14^ in pneumonia. Meta-analyses however, are inconclusive as to whether alcohol is a risk factor for acquiring pneumonia^31^, but support alcohol as a risk factor for death due to pneumonia^13^. However, the majority of studies have categorised alcohol consumption into two groups: alcohol abuse versus non-alcohol abuse or high versus low consumption^6–8,10–12,14,17^. Moreover, the threshold above which consumption has been defined as high have been notably elevated, i.e. 200, 300 or even more grams per week, corresponding to 25 to 37.5 UK units^6,8,14,18,32^, far exceeding the 112 g, or 14 units, per week which is currently considered harmful consumption. Gacouin et al.^32^ studied patients admitted to an ICU due to bacterial infection and demonstrated higher mortality in patients with moderate alcohol consumption, defined as 100–350 g per week, compared to patients with alcohol consumption < 100 g per week. Compared to abstainers, Nuorti et al.^6^ found higher incidence of invasive pneumococcal disease in individuals consuming at least 25 drinks of alcohol per week, but not in individuals consuming less than 25 drinks per week. Almirall et al., and Baik et al., both studied a wide spectrum of alcohol consumption, where Almirall et al., found a higher risk of contracting pneumonia among men consuming 40 g of alcohol per day^5^ whereas Baik et al., saw no associations, although their study had relatively few cases of pneumonia with high alcohol consumption. To our knowledge, no previous study has investigated the full spectrum of alcohol consumption with as high resolution as the present study. Compared to moderate alcohol consumption, we found a marginally higher risk of developing and dying due to bacterial infection in individuals with very low alcohol consumption, as well as a more pronounced risk among individuals with high alcohol consumption. This supports theories of nonlinear effects of alcohol on the immune system^3^. This “J-shaped” dose–response association between alcohol consumption and bacterial infection has previously also been shown for alcohol and other outcomes, including all-cause mortality^33^, but its validity has been questioned^34^. It is possible that alcohol abstainers may have a higher prevalence of previous alcohol abuse, or comorbidities or medications contraindicating alcohol consumption^35^. Conversely, a low-to-moderate alcohol consumption may be associated with other factors associated with good health, such as a strong social life^35^. Although our analyses were adjusted for many known confounders, residual confounding can never be fully eliminated in observational studies. Replication using other methodologies, such as Mendelian randomisation, may afford further insights.

Weaknesses of this study include use of a single self-report of alcohol intake at baseline, which is likely to underestimate consumption^36^. However, this would probably attenuate the results, as would death from other alcohol-related diseases. Too few participants reported complete abstinence to permit analyses. Although statistical adjustments were made for several known risk factors for infection, further information on comorbidities would be valuable, as would information on other potential confounders; such as diet, vaccination status, immunosuppression and frailty. Further, information on specific strain of bacteria and resistance pattern would be interesting. Participants in the cohorts are likely more healthy than non-participants, but this affects external, and not internal, validity^37^. Further, unmeasured or unknown confounding can never be excluded in observational research. Strengths of this study include the use of large population-based cohorts, representative of the underlying population with virtually no losses during a very long to follow-up in national registers of high quality.

In conclusion, alcohol consumption has a nonlinear J-shaped dose–response association with the risk of acquiring and dying due to bacterial infection and pneumonia, where moderate consumption between one-half and four UK units per day is not associated with bacterial infection, but both lower and higher consumption is associated with higher risks. It seems that the level of alcohol consumption needed to increase the risk of bacterial infection is higher than what increases the risk of other conditions. This supports the growing evidence that bacterial infection may be considered a lifestyle disease, and, if our findings are replicated, that lifestyle interventions may lower the risk of bacterial infection.

## Supplementary Information

Supplementary Information 1.

Supplementary Information 2.

Supplementary Information 3.

Supplementary Information 4.

Supplementary Information 5.

Supplementary Information 6.

## Data Availability

The dataset underlying the conclusions of this article cannot be publicly shared due to sensitive details. Any researcher with a valid ethical approval can contact the office of the Swedish Infrastructure for Medical Population-based Life-course and Environmental Research at simpler@surgsci.uu.se or via the online application form at www.simpler4health.se to request data.
