# Supplementary material for: Alcohol consumption has a J-shaped association with bacterial infection and death due to infection, a population-based cohort study
Source: Sci Rep. 2025 Mar 1;15:7333. doi: 10.1038/s41598-025-90197-8 (PMC11873035; doi:10.1038/s41598-025-90197-8)
Supplement: Supplementary file 1 — Supplementary Information 1. [file 41598_2025_90197_MOESM1_ESM.pdf]

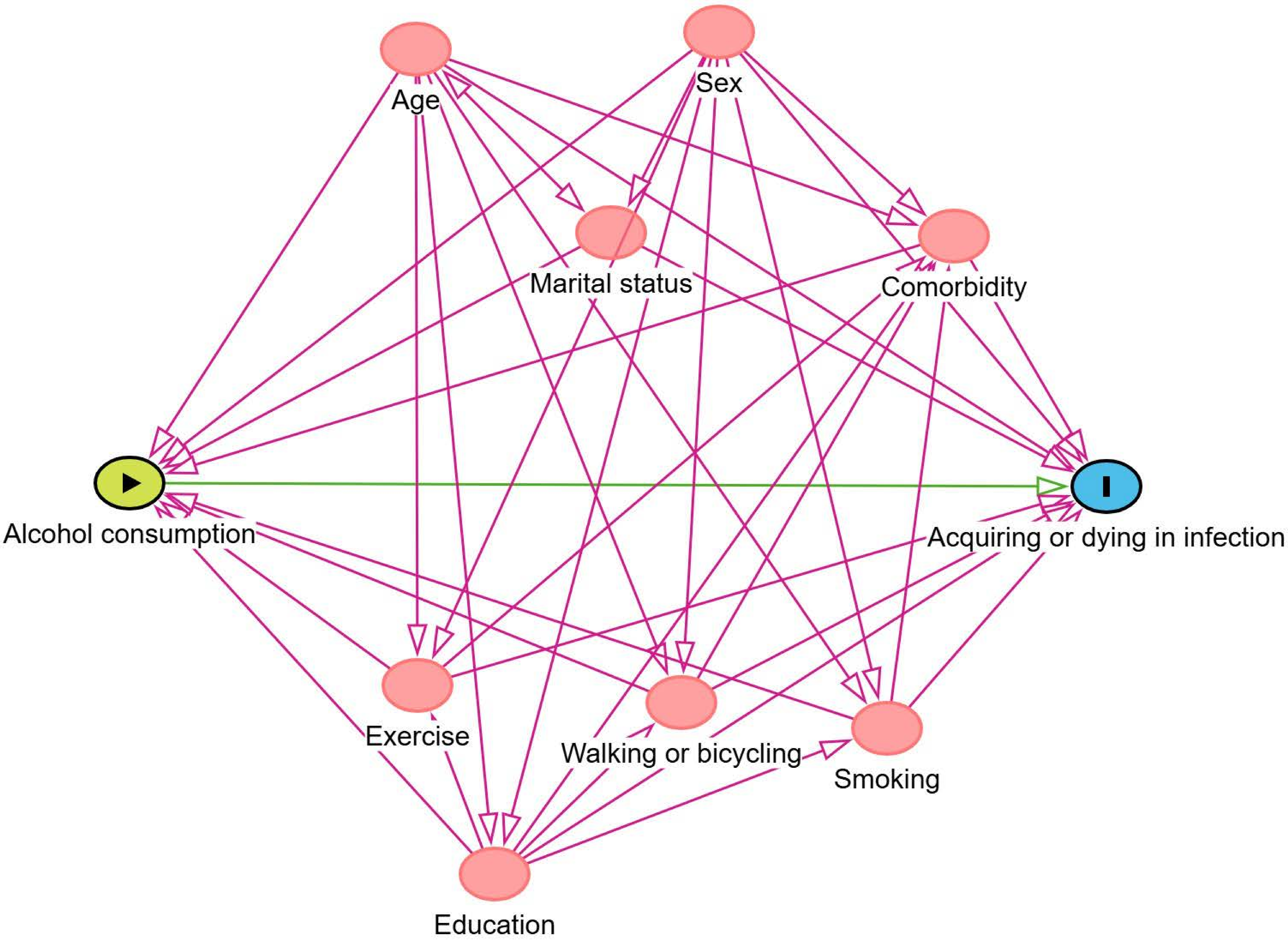

Supplemental Figure 1: Directed Acyclic Graph describing causal assumptions underlying the main analysis.
