# Supplementary material for: Alcohol consumption has a J-shaped association with bacterial infection and death due to infection, a population-based cohort study
Source: Sci Rep. 2025 Mar 1;15:7333. doi: 10.1038/s41598-025-90197-8 (PMC11873035; doi:10.1038/s41598-025-90197-8)
Supplement: Supplementary file 2 — Supplementary Information 2. [file 41598_2025_90197_MOESM2_ESM.pdf]

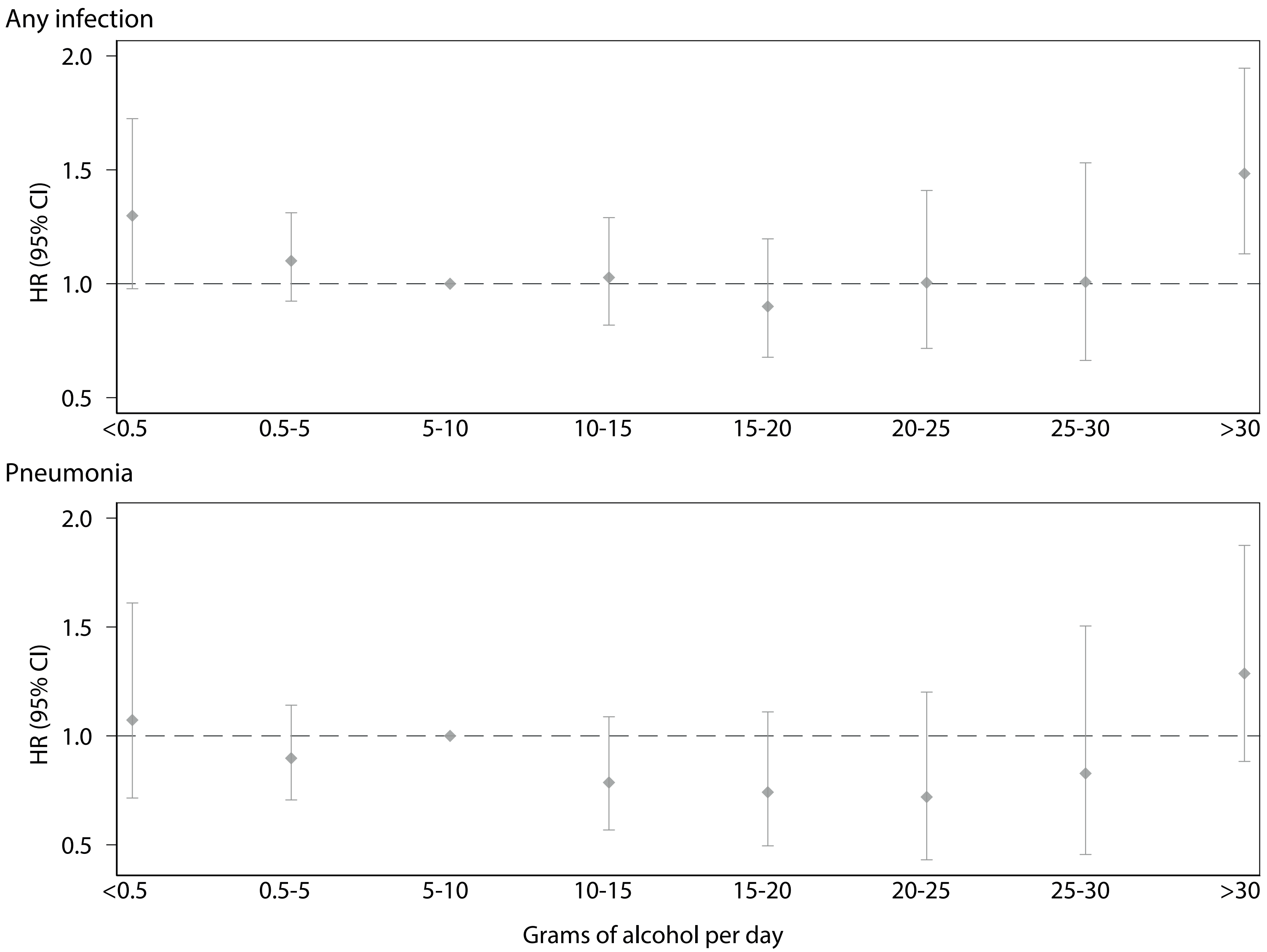

Supplemental Figure 2: Hazard ratio (HR) and 95% confidence intervals (CI) of dying with bacterial infection as underlying cause of death.

Hazard ratio (HR) and 95% confidence interval (CI) of dying due to bacterial infection and pneumonia (light grey diamond), where only the underlying cause of death is considered, by alcohol consumption in grams per day, adjusted for age, sex, exercise, walking or bicycling, education, marital status, smoking status and Charlson's weighted comorbidity index.
