# Supplementary material for: Alcohol consumption has a J-shaped association with bacterial infection and death due to infection, a population-based cohort study
Source: Sci Rep. 2025 Mar 1;15:7333. doi: 10.1038/s41598-025-90197-8 (PMC11873035; doi:10.1038/s41598-025-90197-8)
Supplement: Supplementary file 3 — Supplementary Information 3. [file 41598_2025_90197_MOESM3_ESM.pdf]

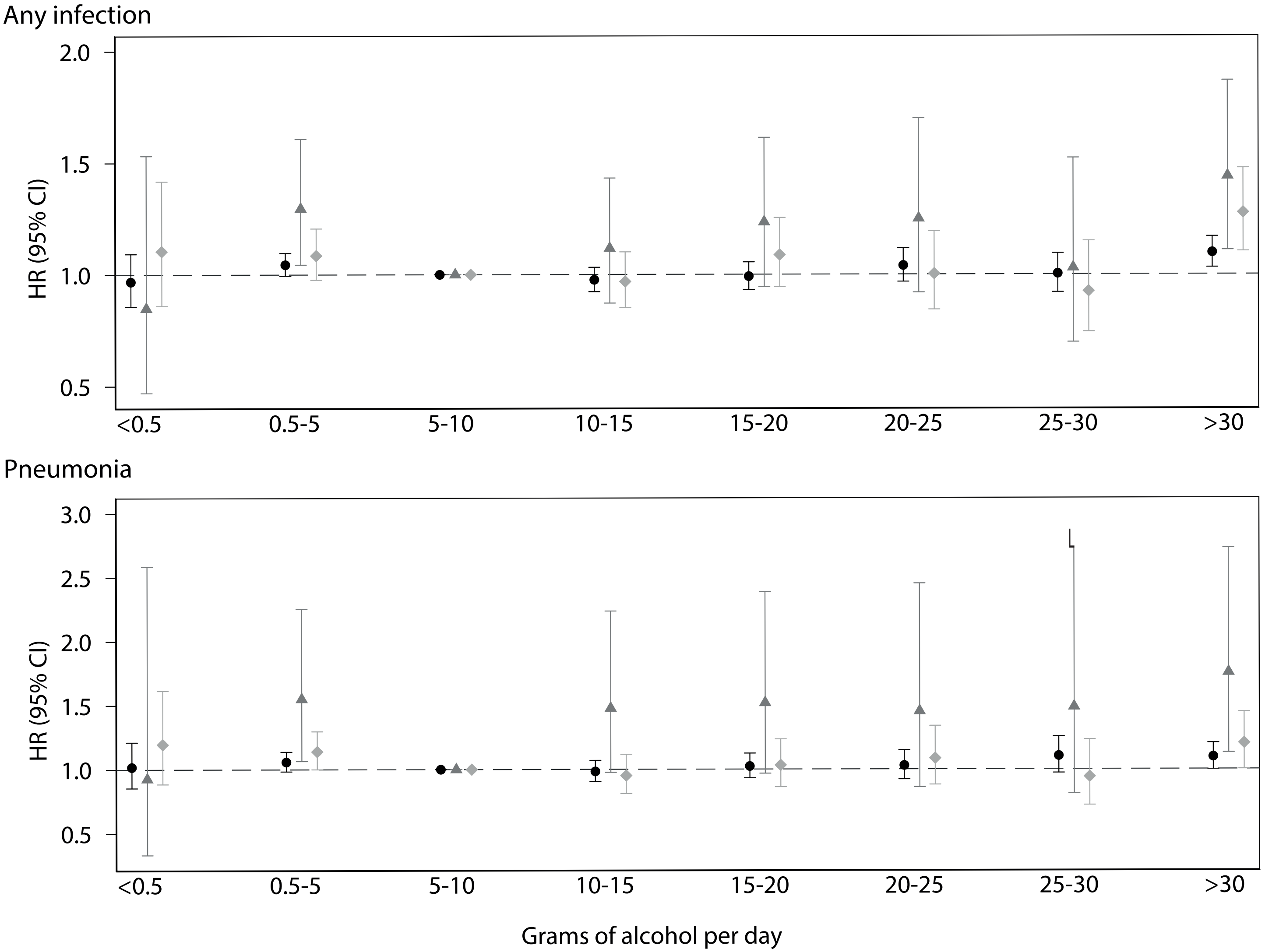


Supplemental Figure 3: Hazard ratio (HR) and 95% confidence intervals (CI) of developing bacterial infection, ICU admission and death adjusted for self-rated health.

Hazard ratio (HR) and 95% confidence interval (CI) of developing bacterial infection (black circle), being admitted to an intensive care unit (grey triangle) and dying due to bacterial infection (light grey diamond) for any bacterial infection (upper panel) and pneumonia (lower panel) by alcohol consumption in grams per day, adjusted for age, sex, exercise, walking or bicycling, education, marital status, smoking status, Charlson's weighted comorbidity index and self-rated health.
