# Supplementary material for: Alcohol consumption has a J-shaped association with bacterial infection and death due to infection, a population-based cohort study
Source: Sci Rep. 2025 Mar 1;15:7333. doi: 10.1038/s41598-025-90197-8 (PMC11873035; doi:10.1038/s41598-025-90197-8)
Supplement: Supplementary file 4 — Supplementary Information 4. [file 41598_2025_90197_MOESM4_ESM.pdf]

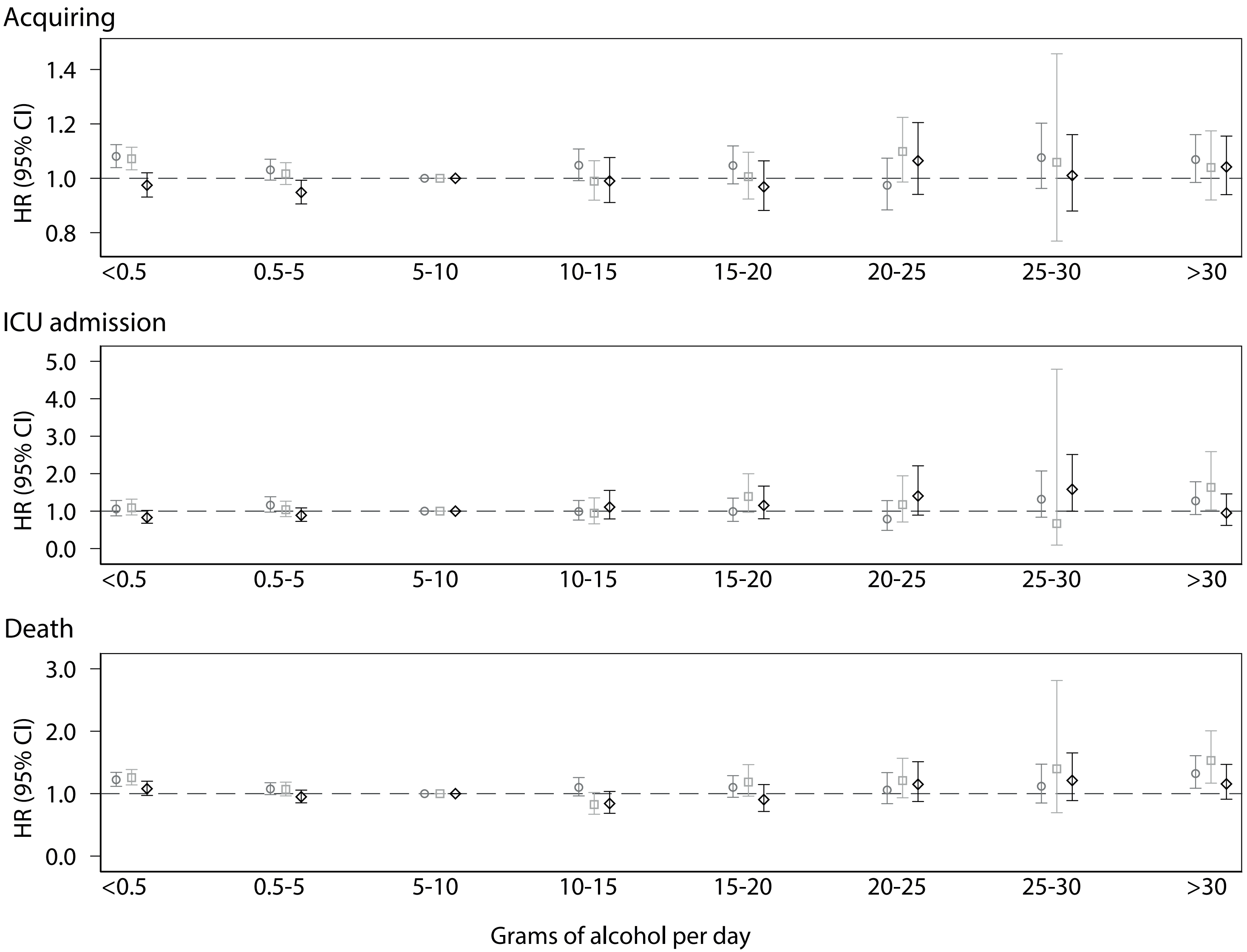

Supplemental Figure 4: Hazard ratio (HR) and 95% confidence intervals (CI) of developing bacterial infection, ICU admission and death by type of alcoholic beverage.
Hazard ratio (HR) and 95% confidence interval (CI) of developing any bacterial infection (upper panel), being admitted to an intensive care unit (middle panel) and dying due to any bacterial infection (lower panel) by consumption alcohol from beer (hollow circle), wine (hollow square) and spirits (hollow diamond) in grams per day, adjusted for age, sex, exercise, walking or bicycling, education, marital status, smoking status and Charlson's weighted comorbidity index.
